# Optimization of Electrical and Mechanical Properties through the Adjustment of Design Parameters in the Wet Spinning Process of Carbon Nanotube/Polyvinylidene Fluoride Fibers Using Response Surface Methodology

**DOI:** 10.3390/polym15143090

**Published:** 2023-07-19

**Authors:** Chan-Woong Choi, Ji-Won Jin, Ki-Weon Kang

**Affiliations:** 1 Department of Green Mobility R&D, Jeonbuk Institute of Automotive Convergence Technology, Gunsan 54158, Republic of Korea; cwchoi@jiat.re.kr; 2 Korea Testing Laboratory, System Verification and Validation Center, Seoul 08389, Republic of Korea; jinjiwon@ktl.re.kr; 3 Department of Mechanical Engineering, Kunsan National University, Gunsan 54150, Republic of Korea

**Keywords:** analysis of variance, CNT/PVDF fiber, carbon nanotube, central composite design (CCD), electrical conductivity, multiple properties, polyvinylidene fluoride, response surface method (RSM), tensile strength, wet spinning

## Abstract

The optimal process conditions for fabricating carbon nanotube (CNT)/polyvinylidene fluoride (PVDF) fibers with varying properties using a wet spinning process were experimentally determined. A dope solution was prepared using multi-walled nanotubes, PVDF, and dimethylacetamide, and appropriate materials were selected. Design parameters affecting the chemical and physical properties of CNT/PVDF fibers, such as bath concentration, bath temperature, drying temperature, and elongation, were determined using a response surface method. The wet-spinning conditions were analyzed based on the tensile strength and electrical conductivity of the fibers using an analysis of variance and interaction analysis. The optimized process conditions for fabricating CNT/PVDF fibers with different properties were derived and verified through fabrication using the determined design parameters.

## 1. Introduction

Carbon nanotubes (CNTs) have attracted significant research interest, owing in part to their high mechanical strength and electrical conductivity. They have been widely used in various fields to overcome the limitations of composite materials and provide enhanced functionality [1,2,3,4,5]. Recently, CNTs have been added to polymers to create fibers that can reinforce strength and function as sensors. Due to the benefits of these CNT composite fibers, including superior strength compared to conventional fibers, high electrical conductivity, and ease of handling, numerous studies have been conducted to characterize their properties. Su et al. [6] fabricated CNT composite fibers by wet-spinning multiwalled nanotube (MWNT)/polyvinyl alcohol/gelatin. A remarkable increase in tensile strength of the PVA/gelatin fibers was achieved by adding a small amount of CNTs. Mukai et al. [7] presented a manufacturing method for polymer-free CNT fibers, which exhibited high electrical conductivity (14,284 ± 169 S/cm) and tensile strength (887 ± 37 MPa). The method involved the utilization of an organic coagulating solvent and subsequent stretching to align the CNTs within the fiber. Glauß et al. [8] fabricated CNT composite fibers with a specific resistivity of 0.6 Ωm by melt spinning MWNT/PVDF/polypropylene. Mirbaha et al. [9] studied wet spinning of PAN and PAN/CNT fibers and the effect of different shear and stretching conditions on the structural, mechanical, and electrical properties of as-spun fibers. The results showed that at different shear rates, the mechanical strength, Young’s modulus, and strain at break of freely spun PAN/CNT fibers improved up to 20% compared to PAN fibers due to possible formation of an interface between the polymer and CNTs. Kang et al. [10] proposed a performance stabilization process aimed at maintaining the reliability of CNT/PVDF fibers. They applied this process to wet spinning to manufacture and evaluate CNT/PVDF fibers with enhanced reliability. In this study, the bath concentration, bath temperature, drying temperature, and elongation during wet spinning were identified as key process parameters that impact the performance of the CNT/PVDF fibers. Alexopoulos et al. [11,12] synthesized CNT fibers by the wet spinning method and embedded them in glass-fiber-reinforced plastic (GFRP) composites, and reported their piezoresistive sensitivity and damage detection characteristics. Similar to previous studies, these investigations focused on fiber fabrication, with an emphasis on the properties of the CNT composite fibers, such as tensile strength and electrical conductivity. The technology of CNT composite fibers is expected to advance numerous industries and create new markets. To realize this objective, composite fibers must be fabricated with specific properties, depending on their intended application [13,14,15,16,17].

To fabricate fibers with the desired properties, numerous studies have investigated their mechanical and electrical properties. Eun et al. [18] fabricated multiwalled carbon nanotube (MWCNT)/PVDF fibers using electrospinning and investigated the effects of experimental parameters on the mechanical properties. They confirmed that the tensile strength increased by approximately 60% compared to the initial condition when the MWCNT content was 0.008 wt%. Chinnappan et al. [19] fabricated PAN/MWCNT/Cu fibers using electrospinning and evaluated their electrical conductivity for MWNT contents of 0, 0.1, 0.3, and 0.5 wt%. Based on this investigation, they identified the relationship between the MWNT content and electrical conductivity. Mercader et al. [20] produced PVA/MWNT fibers using wet spinning and examined Young’s modulus for fiber elongations of 0%, 75%, 160%, and 200% in a hot elongation process. Based on this investigation, they established a relationship between the elongation of PVA/MWNT fibers and Young’s modulus. In general, most studies aim to improve the mechanical and electrical properties of CNT composite fibers by adjusting the content of the materials that constitute the fibers or the post-treatment process. CNT composite fibers are mainly used for structural reinforcement or as sensors. Thus, although it is important to fabricate fibers with predetermined properties, few relevant studies have investigated this aspect.

The response surface method (RSM) is a useful approach for minimizing repetitive analyses, evaluating interactions between process variables, and optimizing process conditions when there are several variables that affect mechanical and electrical properties, such as in the wet spinning process. RSM was first developed in the field of statistics by Box and Wilson in the 1950s and has since been widely applied in various fields to construct response surfaces and optimize process conditions [21]. For example, Rigotti et al. applied RSM to optimize the process parameters affecting elastic modulus and strain at break in the drawing process of bio-derived polylactide/poly(dodecylene furanoate) fibers using wet spinning [22]. Oroume et al. used RSM to optimize spinning conditions and wet spinning to improve the tensile strength of lignin/polyacrylonitrile carbon fiber precursors [23]. Although RSM is widely used in various fields, studies applying this method to optimize the mechanical and electrical properties of CNT composite fibers are still lacking.

In this study, our objective was to optimize the wet spinning process to achieve multiple properties, including tensile strength and electrical conductivity, in CNT/PVDF fibers. In our previous study [10] on CNT/PVDF fibers, we employed a specific fabrication method and evaluation method. To prepare the fibers, a dope solution was created using MWNT (3 wt%) and PVDF, dissolved in dimethylacetamide (DMAC). The bath concentration, bath temperature, drying temperature, and elongation were selected as the four design parameters influencing the fibers’ tensile strength and electrical conductivity. We determined the experimental conditions using the response surface method (RSM), with tensile strength and electrical conductivity set as the objective functions. We evaluated the fibers’ properties under these conditions, and confirmed their significance through analysis of variance. We derived regression equations for the tensile strength and electrical conductivity based on the four design parameters and conducted surface analysis to define their interactions. Finally, we improved the fibers’ tensile strength and electrical conductivity using the regression equations and established optimized wet spinning process conditions that could achieve multiple physical properties.

## 2. Fabrication of CNT/PVDF Composite Fibers Using Wet Spinning

### 2.1. Material Selection and Fabrication Process

**Figure 1:**
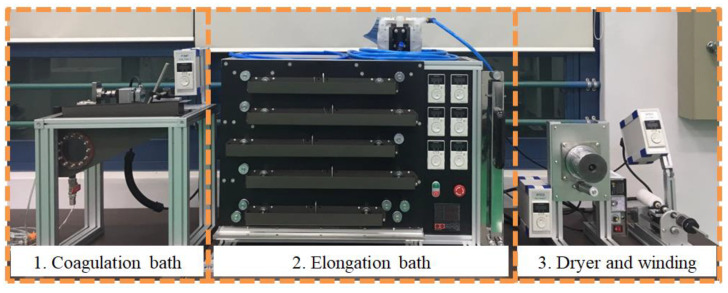
Wet-spinning process for the production of CNT/PVDF fibers.

In our previous study [10] on CNT/PVDF fibers, we employed specific material selection and manufacturing process methods. Specifically, to produce highly reliable CNT/PVDF fibers, we utilized three processes: dope solution stabilization, cylinder pressure application, and room temperature drying of CNT/PVDF fibers. These processes were implemented to minimize the presence of voids within the fibers. To produce CNT/PVDF fibers through wet spinning, a dope solution comprising MWNT (K-Nanos 100) (Table 1), PVDF powder (Kynar^®^ 761, Arkema, Colombes, France) (Table 2), and N, N-DMAC (SAMCHUN Chemical Co., Seoul, Republic of Korea) (Table 3) was utilized. The ratio of the dope solution components was MWNT:PVDF:DMAC = 0.6:20:79.4. Figure 1 illustrates the wet spinning system (Dissol, Jeonju, Republic of Korea) used in the study. The dope solution was extruded into a coagulation bath via a gear pump and mono-hole nozzle. It was subsequently passed through washing and elongation baths to enhance the orientation and mechanical properties of the CNTs. The fibers were then dried using a heating roller to remove moisture from both the inside and outside of the fibers.

### 2.2. Determination of Design Parameters Using RSM

Process conditions play a crucial role in the fabrication of CNT/PVDF fibers using wet spinning. In a previous study on the selection of appropriate wet spinning process conditions for specific design parameters, Baojun et al. [24] investigated the effect of voids inside the fibers caused by the diffusion rate of the solvent and the firming agent on the mechanical properties. They found that solidification conditions, such as bath concentration and temperature, can affect the diffusion rate. Kun et al. [25] produced PVDF fibers using a wet spinning process and investigated the influence of design parameters, such as elongation and thermal curing temperature, on the tensile strength of the fibers in a continuous wet spinning system. Based on these studies, the bath concentration, bath temperature, drying temperature, and elongation were selected as process parameters that can affect the properties of continuous fibers.

As multiple design parameters were expected to affect the mechanical and electrical properties of the fibers fabricated via the wet spinning process, the issue of exploring multiple variables in as few experiments is possible and was addressed by means of proper central composite design (CCD) [26]. ANOVA was used to detect the significance of the variables, and multivariate quadratic regression was used to quantitatively optimize the fiber fabrication. To optimize the objective functions, namely, the tensile strength and electrical conductivity of the wet spinning process, a commercial software program, MINITAB 17.3.1ver [27], was used. Table 4 presents the design matrix of the CCD for the four parameters. The results indicate that the total number of experiments for the process conditions was 31, of which seven experiments were repeated to improve the reliability of the results. Based on the determined wet spinning conditions, CNT/PVDF fibers were fabricated.

## 3. CNT/PVDF Fibers

### 3.1. Characterization

The CNT/PVDF fibers were characterized using the three techniques proposed by Kang [10]. First, scanning electron microscopy (SU8220, Hitachi, Japan) was used to image the cross-sectional area inside the CNT/PVDF fibers that affects their mechanical properties. Additionally, the mechanical strength of the fibers was calculated using ASTM D3379 [28]. For each wet spinning condition, 30 specimens were taken from the 30 m, 60 m, and 90 m sections. Both ends of each fiber were fixed with adhesive to a paper guide, with a gauge length of 25 mm, to prepare a tensile specimen of CNT/PVDF fiber. The tensile test was performed at a speed of 2 mm/min.

Finally, the electrical conductivity of the CNT/PVDF fibers was measured following the guidelines outlined in IEC 60093 [29] to verify their electrical characteristics. The electrical conductivity of the CNT/PVDF fibers was measured to determine their electrical properties. Each fiber was mounted on a fixing jig to maintain a gauge length of 1 cm under room temperature atmospheric conditions. A voltage of 10 V was applied for 10 s, and then the resistance was measured after the voltage was cut off. The calculated resistance was then converted into electrical conductivity.

### 3.2. Evaluation via RSM under Different Wet Spinning Conditions

**Figure 2:**
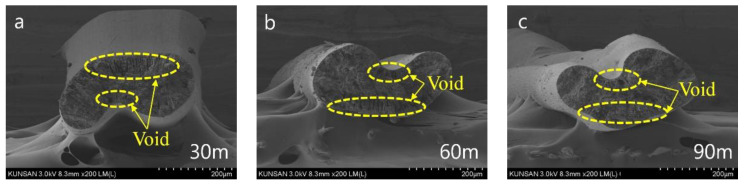
Fiber cross-section image of Case 7 (*x*_1_:*x*_2_:*x*_3_:*x*_4_ = 10:60:120:200) according to fiber location: (**a**) 30 m, (**b**) 60 m, (**c**) 90 m.

**Figure 3:**
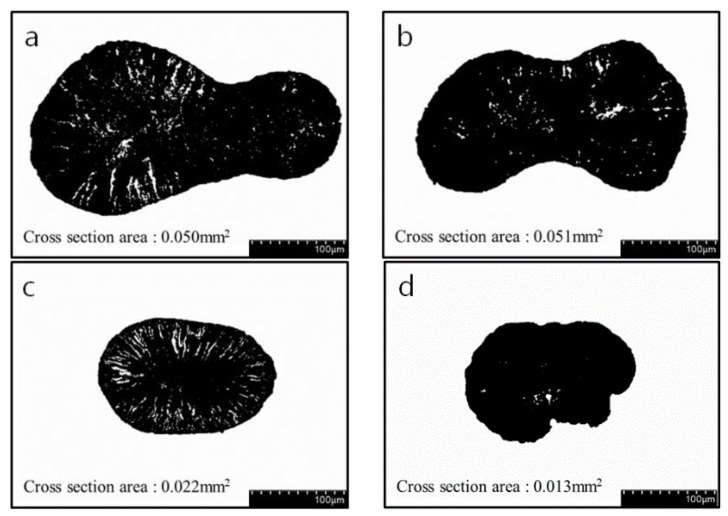
Cross section area of RSM conditions in CNT/PVDF fibers: (**a**) Case 2 (*x*_1_:*x*_2_:*x*_3_:*x*_4_ = 30:40:100:200), (**b**) Case 5 (*x*_1_:*x*_2_:*x*_3_:*x*_4_ = 10:40:120:200), (**c**) Case 10 (*x*_1_:*x*_2_:*x*_3_:*x*_4_ = 30:40:100:400), (**d**) Case 22 (*x*_1_:*x*_2_:*x*_3_:*x*_4_ = 20:50:130:300).

The CNT/PVDF fibers were spun according to the wet spinning conditions obtained using RSM. Thirty specimens were collected from each section of the fibers (30, 60, and 90 m) to perform tensile and electrical conductivity measurements. Before conducting the tensile test, the fiber’s cross section was captured using FE-SEM (field emission scanning electron microscopy). Representative results of case 7 (*x*_1_:*x*_2_:*x*_3_:*x*_4_ = 10:60:120:200) are presented in Figure 2. SEM fiber samples were obtained at 30 m, 60 m, and 90 m positions, and the fiber’s shape and void location remained consistent regardless of the length variation. Next, the cross-sectional area of the fibers was measured before conducting the tensile test, and representative results are shown in Figure 3. ImageJ 1.53ver [30] was utilized for the analysis of the cross-sectional area. The cross-sectional area was measured using one specimen for each measurement section, and it was confirmed that the diameter of the cross-sectional area differed depending on the spinning conditions. Table 5 summarizes the tensile test results of 90 specimens according to the spinning conditions. The average tensile strength for cases 1 to 31 ranged from 18.94 to 63.78 MPa, indicating that the deviation of the tensile strength was constant for each case. The maximum tensile strength was determined to be 63.78 MPa for the conditions used in case 22 (*x*_1_:*x*_2_:*x*_3_:*x*_4_ = 20:50:130:300), and the minimum tensile strength was 18.94 MPa for the conditions used in case 2 (*x*_1_:*x*_2_:*x*_3_:*x*_4_ = 30:40:100:200). The tensile test results demonstrated that the tensile strength differed by more than three times depending on the design parameters.

The electrical conductivity measurements were conducted using specimens obtained similarly to the tensile strength specimens. The results are presented in Table 6. For cases 1–31, the average electrical conductivity ranged from 4.35 × 10^−5^ to 2.00 × 10^−6^ S/cm, indicating that the deviation of the electrical conductivity was constant for each case. The maximum electrical conductivity was found to be 4.35 × 10^−5^ S/cm for case 5 (*x*_1_:*x*_2_:*x*_3_:*x*_4_ = 10:40:120:200), and the minimum was 2.00 × 10^−6^ S/cm for case 10 (*x*_1_:*x*_2_:*x*_3_:*x*_4_ = 30:40:100:400). The coefficient of variation (COV) values confirmed that the deviation of the electrical conductivity was very small, ranging from 2.19 × 10^−2^ to 5.17 × 10^−2^. In particular, the maximum tensile strength was calculated for case 22 (*x*_1_:*x*_2_:*x*_3_:*x*_4_ = 20:50:130:300), and the maximum electrical conductivity was calculated for case 5 (*x*_1_:*x*_2_:*x*_3_:*x*_4_ = 10:40:120:200). These calculations were performed under different process conditions. Therefore, it was found that both the tensile strength and the electrical conductivity were influenced by the design variables.

**Figure 4:**
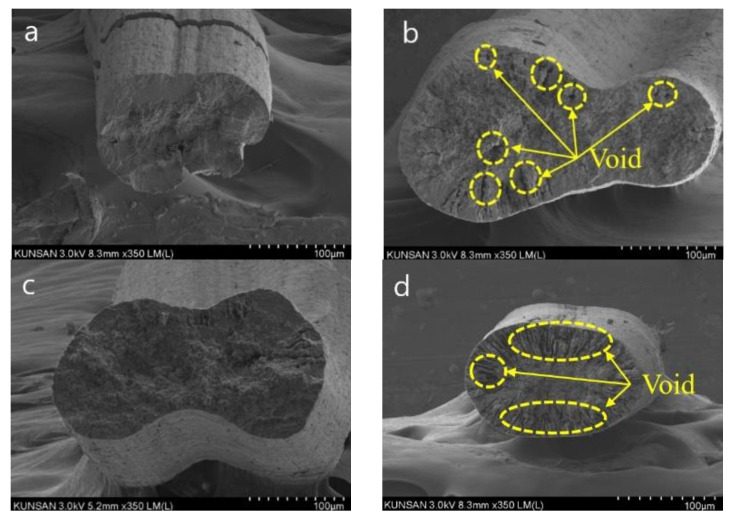
SEM images of CNT/PVDF fibers for the RSM conditions: (**a**) Case 22 (*x*_1_:*x*_2_:*x*_3_:*x*_4_ = 20:50:130:300), (**b**) Case 2 (*x*_1_:*x*_2_:*x*_3_:*x*_4_ = 30:40:100:200), (**c**) Case 5 (*x*_1_:*x*_2_:*x*_3_:*x*_4_ = 10:40:120:200), and (**d**) Case 10 (*x*_1_:*x*_2_:*x*_3_:*x*_4_ = 30:40:100:400).

The FE-SEM imaging results shown in Figure 4 provide insights into the interaction of the inner geometry of the fibers with their tensile strength and electrical conductivity. It is observed that the internal structure of the fibers has a significant impact on their properties. Case 22 (Figure 4a), which had the maximum tensile strength, had a dense internal structure with a small number of internal voids. In contrast, Case 2 (Figure 4b), which had the lowest tensile strength, had a decreased tensile strength due to the large number of voids, as indicated by the yellow circle. This indicates that a dense internal structure with fewer voids is required to improve the tensile strength of the fibers. Similarly, Case 5 (Figure 4c), which had the maximum electrical conductivity, had a dense CNT network inside the fibers due to the dense internal structure with fewer voids. In contrast, Case 10 (Figure 4d), which had the lowest electrical conductivity, as indicated by the yellow circle, had voids created inside the fiber due to the rapid escape of DMAC. Because of this, the CNT network inside the fiber was destroyed and the electrical conductivity was lowered.

Therefore, the internal density and number of voids significantly affect the tensile strength and electrical conductivity of the CNT/PVDF fibers. These results demonstrate that adjusting the design parameters can reduce voids and improve the electrical and physical properties of the fibers. By optimizing the spinning conditions to achieve a dense internal structure with fewer voids, the tensile strength and electrical conductivity of the CNT/PVDF fibers can be improved.

## 4. Optimization of Multiple Properties of CNT/PVDF Fibers

### 4.1. Analysis of Variance (ANOVA)

ANOVA was carried out to assess the significance of both tensile strength and electrical conductivity of the CNT/PVDF fibers. Table 7 presents the ANOVA results, which confirm the significance of the second-order regression model for the tensile strength with respect to four design parameters.

The *p*-value represents the probability of obtaining a difference between the observed value and the hypothesized value due to random error, with a value lower than 0.05 indicating the significance of the corresponding parameter. Upon examining the coefficients of the estimated regression model, the values of the first-order terms were found to be 0.05 or less, confirming their significance. Additionally, for the second-order and interaction terms, non-significant coefficients with a *p*-value greater than 0.05 were combined as error terms. The coefficient of determination of the estimated regression equation was 87.50%, making it suitable for estimating the tensile strength of the CNT/PVDF fibers. Therefore, a second-order regression Equation (1) for estimating tensile strength using the four parameters was derived.

(1) $$\begin{array}{ll} y_{Tensilestrength}= & -\left(7.48\times 10\right)-\left(9.00\times 10^{-1}\right)x_{1}+3.58x_{2}-\left(7.25\times 10^{-1}\right)x_{3}+\left(1.60\times 10^{-1}\right)x_{4}\\ & -\left(5.85\times 10^{-2}\right){x_{1}}^{2}-\left(4.79\times 10^{-2}\right){x_{2}}^{2}-\left(2.10\times 10^{-4}\right){x_{4}}^{2}+\left(3.24\times 10^{-2}\right)x_{1}x_{3}\\ & +\left(1.51\times 10^{-2}\right)x_{2}x_{3} \end{array}$$

Table 8 presents the ANOVA results for the second-order regression model that used electrical conductivity as the objective function for the four parameters. Additionally, for the first-order, second-order, and interaction terms, non-significant coefficients with a *p*-value greater than 0.05 were combined as error terms. It was confirmed that an error occurred in the case of the error term since seven repeated experiments were conducted for cases 25 to 31, and their results are shown in Table 8. In this case, the coefficient of determination of the estimated regression equation was 80.12%, which is suitable for estimating the electrical conductivity of the CNT/PVDF fibers. Therefore, a second-order regression Equation (2) was derived for estimating electrical conductivity using the four parameters.

(2) $$\begin{array}{ll} y_{Electricalconductivity}\;= & -\left(4.56\times 10^{-6}\right)+\left(5.32\times 10^{-8}\right)x_{1}+\left(2.48\times 10^{-7}\right)x_{2}\\ & +(2.41\times 10^{-7})x_{3}-(4.52\times 10^{-8})x_{4}+(3.04\times 10^{-9}){x_{2}}^{2}\\ & -(5.70\times 10^{-9})x_{1}x_{2}+(3.80\times 10^{-10})x_{1}x_{4}-(3.04\times 10^{-9})x_{2}x_{3} \end{array}$$

The derived second-order regression equations can be used to estimate tensile strength and electrical conductivity.

### 4.2. Response Surface Analysis

**Figure 5:**
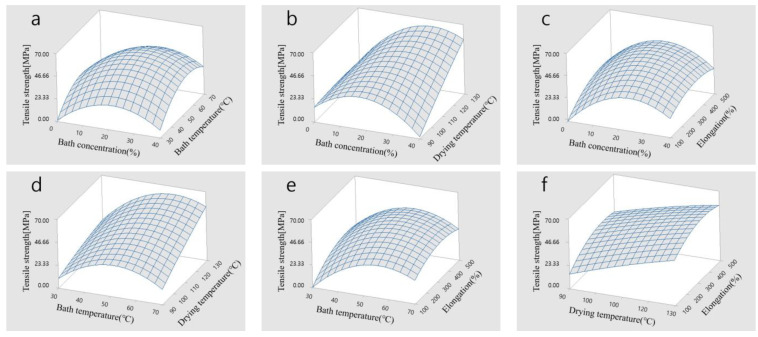
Surface view of tensile strength: (**a**) bath concentration (%) and bath temperature (°C), (**b**) bath concentration (%) and drying temperature (°C), (**c**) bath concentration (%) and elongation (%), (**d**) bath temperature (°C) and drying temperature (°C), (**e**) bath temperature (°C) and elongation (%), and (**f**) drying temperature (°C) and elongation (%).

To investigate the impact of the wet spinning design parameters on the objective functions of electrical conductivity and tensile strength, a surface analysis was conducted. Figure 5a–f present the results of the surface analysis for tensile strength. In Figure 5a, it is observed that the highest tensile strength was achieved when the bath concentration was 20% and the bath temperature was 50 °C. This is because the bath concentration and temperature have an influence on the reduction rate of DMAC in the dope solution, which affects the formation of voids in the fibers.

In Figure 5b, the bath concentration that improved the tensile strength was similar to that obtained in Figure 5a, but the tensile strength increased as the drying temperature increased. This is because the mechanical properties of the fibers improve as PVDF polymers are oriented in the fiber direction. Moreover, in Figure 5c, the highest tensile strength was achieved at a bath concentration of 20% and elongation of 300%. In Figure 5d, the tensile strength increased as the drying temperature increased at a bath temperature of 50 °C. This is because the internal density of the fibers increased as the drying temperature increased. In Figure 5e, the maximum tensile strength was achieved at a bath concentration of 20% and elongation of 300%. In Figure 5f, the tensile strength improved as the drying temperature and elongation increased. These findings confirm that the design parameters, including the bath concentration, bath temperature, drying temperature, and elongation, have combined effects on the tensile strength, and all of them should be considered simultaneously to enhance this parameter.

**Figure 6:**
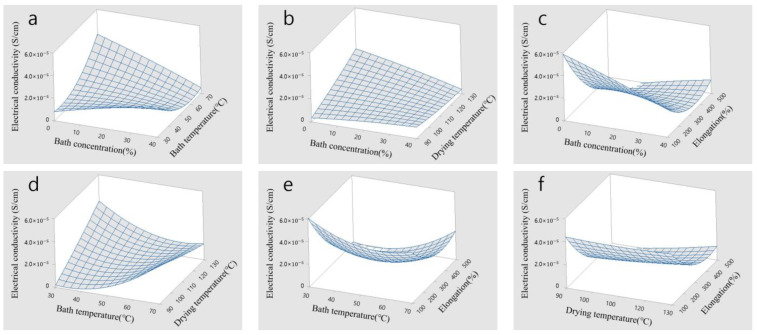
Surface view of electrical conductivity: (**a**) bath concentration (%) and bath temperature (°C), (**b**) bath concentration (%) and drying temperature (°C), (**c**) bath concentration (%) and elongation (%), (**d**) bath temperature (°C) and drying temperature (°C), (**e**) bath temperature (°C) and elongation (%), and (**f**) drying temperature (°C) and elongation (%).

Figure 6a–f show the surface view of the samples used for investigating electrical conductivity. Based on the graphs, electrical conductivity was high when there was an inverse relationship between the bath concentration and bath temperature, as shown in Figure 6a,b. This is because the cross-sectional area of the CNT/PVDF fibers decreases as the bath concentration increases, and the solidification rate of the fibers increases as the bath temperature rises. In Figure 6c, it was confirmed that changes in elongation had a greater effect on the change in electrical conductivity than changes in the bath concentration. Electrical conductivity increases as elongation decreases because an increase in elongation reduces the cross-sectional area of the fibers, which is related to the CNT network inside the CNT/PVDF fibers. In Figure 6d, the effect of the drying temperature was significant when the bath concentration was low. The electrical conductivity improved as the initial cross-sectional area was maintained by the rapid solidification of the CNT/PVDF fibers at low concentrations, and the density of the fibers increased at high drying temperatures. Figure 6e shows the changes in the bath concentration and elongation. There was negligible change in the bath temperature, and the electrical conductivity varied depending on the elongation. This was associated with the change in the CNT network caused by the change in the cross-sectional area of the CNT/PVDF fibers. Figure 6f shows the changes in the drying temperature and elongation. There was almost no change in the drying temperature, and the electrical conductivity varied depending on the elongation. This is also attributed to the change in the CNT network caused by the change in the cross-sectional area of the CNT/PVDF fibers. These results indicate that the design parameters (i.e., bath concentration, bath temperature, drying temperature, elongation) must be simultaneously considered to improve electrical conductivity.

### 4.3. Optimization of Multiple Properties

In order to apply CNT/PVDF fibers in the industry, it is necessary to be able to fabricate them with a range of different properties, such as tensile strength and electrical conductivity. Equation (3) represents an optimization equation for the fabrication of CNT/PVDF fibers with desired properties. Equations (4)–(7) define the ranges of the design parameters, Equations (8)–(10) define the ranges of weights for the tensile strength and electrical conductivity, Equation (11) defines the range of the tensile strength, and Equation (12) defines the range of the electrical conductivity. The spinning conditions required to optimize multiple properties based on these equations were calculated, and the results are shown in Table 9. The tensile strength and electrical conductivity were predicted using the regression Equations (1) and (2) obtained in Section 4.1, and compared with the test values (Table 10).

Find *x*_1_, *x*_2_, *x*_3_, *x*_4_

To maximize tensile strength and electrical conductivity

Object function max

(3) $$\alpha_{1}G_{1}\left(x_{1},\;x_{2},\;x_{3},\;x_{4}\right)+\alpha_{2}G_{2}(x_{1},\;x_{2},\;x_{3},\;x_{4})$$

Subjected to

(4) $$0\leq x_{1}\leq 40$$

(5) $$30\leq x_{2}\leq 70$$

(6) $$90\leq x_{3}\leq 130$$

(7) $$100\leq x_{4}\leq 500$$

(8) $$0\leq \alpha_{1}\leq 1$$

(9) $$0\leq \alpha_{2}\leq 1$$

(10) $$\alpha_{1}+\alpha_{2}=1$$

(11) $$G_{1}(x_{1},\;x_{2},\;x_{3},\;x_{4})>0$$

(12) $$G_{2}\left(x_{1},\;x_{2},\;x_{3},\;x_{4}\right)>0$$

where,

$x_{1}$: Bath concentration (%)
$x_{2}$: Bath temperature (°C)
$x_{3}$: Drying temperature (°C)
$x_{4}$: Elongation (%)
$\alpha_{1}$: Weight of tensile strength
$\alpha_{2}$: Weight of electrical conductivity
$G_{1}(x_{1},\;x_{2},\;x_{3},\;x_{4})$ = Function of tensile strength
$G_{2}(x_{1},\;x_{2},\;x_{3},\;x_{4})$ = Function of electrical conductivity

**Figure 7:**
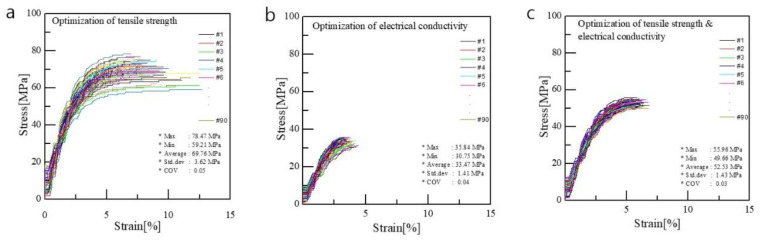
Results of tensile tests for optimization conditions: (**a**) optimization of tensile strength (*x*_1_:*x*_2:_*x*_3:_*x*_4_ = 27.88:54.48:130:382.83), (**b**) optimization of electrical conductivity (*x*_1_:*x*_2:_*x*_3:_*x*_4_ = 20.25:39.87:130:100), and (**c**) optimization of tensile strength and electrical conductivity (*x*_1_:*x*_2:_*x*_3:_*x*_4_ = 37.03:58.27:128.1:100).

**Figure 8:**
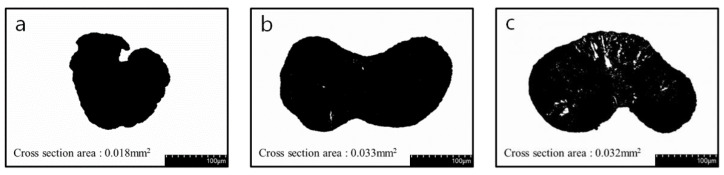
Cross section area of optimization conditions CNT/PVDF fibers: (**a**) optimization of tensile strength conditions (*x*_1_:*x*_2:_*x*_3:_*x*_4_ = 27.88:54.48:130:382.83), (**b**) optimization of electrical conductivity conditions (*x*_1_:*x*_2:_*x*_3:_*x*_4_ = 20.25:39.87:130:100), and (**c**) optimization of tensile strength & electrical conductivity (*x*_1_:*x*_2:_*x*_3:_*x*_4_ = 37.03:58.27:128.1:100).

The design parameters required to optimize the tensile strength were calculated using Equations (3)–(12) to optimize multiple properties. In this process, a weight of 1 was assigned to the tensile strength ($\alpha_{1}$) in Equation (3). The bath concentration (*x*_1_) at 27.88%, bath temperature (*x*_2_) at 54.48 °C, drying temperature (*x*_3_) at 130 °C, and elongation (*x*_4_) at 382.83% were selected as design parameters to minimize voids inside the CNT/PVDF fibers and increase the internal density. The predicted value of the tensile strength was calculated using the parameters mentioned above in Equation (1), and it was found to be 68.18 MPa. To verify the predicted tensile strength, CNT/PVDF fibers were fabricated using the optimized spinning conditions, and tensile tests were conducted. The calculated value was 69.759 MPa (Figure 7a), taking into account the cross-sectional area of the fibers (0.018 ${mm}^{2}$; Figure 8a). The error ((predicted value − experimental value)/predicted value × 100) was 2.3%.

**Figure 9:**
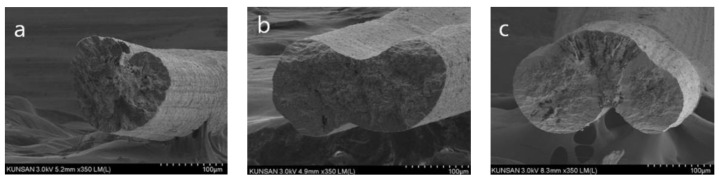
SEM cross section image of optimization conditions: (**a**) optimization of tensile strength conditions (*x*_1_:*x*_2:_*x*_3:_*x*_4_ = 27.88:54.48:130:382.83), (**b**) optimization of electrical conductivity conditions (*x*_1_:*x*_2:_*x*_3:_*x*_4_ = 20.25:39.87:130:100), and (**c**) optimization of tensile strength and electrical conductivity (*x*_1_:*x*_2:_*x*_3:_*x*_4_ = 37.03:58.27:128.1:100).

The optimized spinning conditions for high tensile strength were established, given that the coefficient of determination (87.50%) of the tensile strength regression equation calculated using ANOVA was satisfied. The tensile strength was increased because the number of voids inside the fibers decreased, as confirmed by the SEM image of the fiber cross section shown in Figure 9a. This improvement was attributed to the fact that the exchange rate of DMAc between the dope liquid and the water in the bath was reduced, resulting in an improved orientation of the MWCNTs inside the fiber and an increased density of the structure during the elongation process.

Optimizing the weight of electrical conductivity ($\alpha_{2}$) in Equation (3) resulted in the selection of the bath concentration *x*_1_ (20.25%), bath temperature *x*_2_ (39.87 °C), drying temperature *x*_3_ (130 °C), and elongation *x*_4_ (100%) to improve the network between the CNTs while minimizing the voids inside the CNT/PVDF fibers. Regression Equation (1) was used with these values to calculate the predicted value of tensile strength, which was found to be 32.78 MPa. The experimental value was calculated to be 33.47 MPa (Figure 7b) based on the cross-sectional area of the fibers (0.033 mm^2^; Figure 8b), with an error of 2.1%. The coefficient of determination for the tensile strength regression equation (87.50%) calculated using ANOVA was satisfied.

Regression Equation (2) was applied to predict the electrical conductivity based on the optimized spinning conditions, resulting in a predicted value of 5.84 × 10^−5^ S/cm. The experimental value for these conditions was determined to be 5.23 × 10^−5^ S/cm, with an error of 11.7%. The electrical conductivity improved mainly because the number of voids was reduced due to the reduction of DMAC, as shown in the SEM image of the fiber cross-section in Figure 9b). This was also attributed to the reduction in fiber elongation owing to the improved network between the CNTs.

The optimization of multiple properties, specifically tensile strength and electrical conductivity, is an important aspect in the development of advanced materials. In this study, we optimized the spinning conditions of CNT/PVDF fibers to enhance both properties simultaneously.

To achieve this, we used a multi-objective optimization approach by assigning weights to the properties of interest in Equation (3). By varying the bath concentration, bath temperature, drying temperature, and elongation, we were able to obtain optimal conditions that led to the densification of the internal structure, reduction of voids, and improved network between the CNTs.

The optimized spinning conditions for the tensile strength and electrical conductivity were achieved by varying the weight ratio of ($\alpha_{1}:\alpha_{2}$) and the values of *x*_1_, *x*_2_, *x*_3_, and *x*_4_ The predicted values for both properties were calculated using regression equations, and the experimental values were obtained through testing. The errors between the predicted and experimental values were less than 30% for both properties, indicating prediction accuracy.

The improvement in the properties of interest was attributed to the reduction in DMAC and the drying temperature, which led to the densification of the internal structure and reduced voids. The SEM images of the fiber cross-sections showed the reduction in voids and the improved network between the CNTs. The multi-objective optimization approach used in this study allowed for the simultaneous enhancement of both tensile strength and electrical conductivity in CNT/PVDF fibers.

## 5. Conclusions

In this study, the wet spinning process was optimized using RSM to improve the tensile strength and electrical conductivity of CNT/PVDF fibers and achieve multi-properties. As a result, a regression equation was derived that can predict the electrical conductivity and tensile strength with over 70% accuracy. This research method enables the optimization of CNT/PVDF fiber properties, reducing the test time and cost of new fiber development. Therefore, the design parameters required to achieve specific CNT/PVDF fiber properties can be calculated or predicted using the proposed wet spinning optimization process based on RSM, enabling the fabrication of fibers with predetermined specifications.

## Figures and Tables

**Table 1:** Specifications of MWNT.

Type	Value
Density (g/cm^3^)	2.6
Young’s modulus (GPa)	940
Poisson’s ratio	0.20
Bundle length (μm)	Ave. 40~50
Diameter (nm)	Ave. 11~13
Carbon purity (%)	~95
Ref.	General MWCNT

**Table 2:** Specifications of PVDF.

Type	Value
Density (g/cm^3^)	1.78
Young’s modulus (GPa)	2
Poisson’s ratio	0.34
Tensile strength, yield (MPa)	35
Elongation at break (%)	20~100
Melting point (°C)	170
Molecular weight (g/mol)	441,000

**Table 3:** Specifications of DMAC.

Type	Value
Linear formula	CH3CON(CH3)2; C4H9NO
Density (g/cm^3^)	0.937
Melting point (°C)	−20
Boiling point (°C)	164~166
Flash point (°C)	66
Assay (%)	99.5
Molecular weight (g/mol)	87.12

**Table 4:** Wet spinning conditions for CNT/PVDF fibers using CCD.

Case	Bath Concentration *x*_1_, (%)	Bath Temperature *x*_2_, (°C)	Drying Temperature *x*_3_, (°C)	Elongation *x*_4_, (%)
1	10	40	100	200
2	30	40	100	200
3	10	60	100	200
4	30	60	100	200
5	10	40	120	200
6	30	40	120	200
7	10	60	120	200
8	30	60	120	200
9	10	40	100	400
10	30	40	100	400
11	10	60	100	400
12	30	60	100	400
13	10	40	120	400
14	30	40	120	400
15	10	60	120	400
16	30	60	120	400
17	0	50	110	300
18	40	50	110	300
19	20	30	110	300
20	20	70	110	300
21	20	50	90	300
22	20	50	130	300
23	20	50	110	100
24	20	50	110	500
25	20	50	110	300
26	20	50	110	300
27	20	50	110	300
28	20	50	110	300
29	20	50	110	300
30	20	50	110	300
31	20	50	110	300

**Table 5:** Results of tensile test for RSM conditions.

Case	Tensile Strength				
	Max (MPa)	Min (MPa)	Average (MPa)	Std. dev (MPa)	COV
1	21.50	15.05	19.15	0.94	0.05
2	19.88	15.10	18.94	0.79	0.04
3	24.63	17.57	22.67	1.24	0.05
4	21.73	18.70	21.26	0.49	0.02
5	26.54	18.88	23.55	1.69	0.07
6	31.23	25.16	28.32	1.37	0.05
7	40.25	20.22	29.03	4.08	0.14
8	61.51	52.91	58.11	1.77	0.03
9	25.47	21.12	23.91	0.87	0.04
10	36.76	21.40	26.51	3.57	0.13
11	38.95	31.24	34.71	1.71	0.05
12	38.32	26.78	33.02	2.14	0.06
13	37.15	27.95	32.81	2.06	0.06
14	48.83	41.66	45.24	1.98	0.04
15	49.71	35.36	42.61	3.03	0.07
16	55.84	36.82	47.46	4.50	0.09
17	22.58	19.32	21.03	0.82	0.04
18	37.95	29.56	34.77	2.00	0.06
19	25.40	19.76	22.88	1.30	0.06
20	46.95	34.31	41.34	2.42	0.06
21	39.46	32.25	35.99	1.63	0.05
22	67.41	50.01	63.78	3.08	0.05
23	40.49	32.43	38.44	1.20	0.03
24	52.39	42.31	47.36	2.25	0.05
25	52.17	45.17	49.50	1.65	0.03
26	50.38	35.60	47.29	1.77	0.04
27	51.91	46.28	48.47	1.44	0.03
28	48.41	42.92	45.35	1.32	0.03
29	50.00	44.51	46.57	1.32	0.03
30	50.88	45.61	47.75	1.35	0.03
31	48.65	42.76	44.91	1.35	0.03

**Table 6:** Results of electrical conductivity test for RSM conditions.

Case	Electrical Conductivity				
	Max (S/cm)	Min (S/cm)	Average (S/cm)	Std. dev (S/cm)	COV
1	2.31 × 10^−5^	1.94 × 10^−5^	2.13 × 10^−5^	1.08 × 10^−6^	5.09 × 10^−2^
2	2.63 × 10^−5^	2.29 × 10^−5^	2.46 × 10^−5^	9.61 × 10^−7^	3.90 × 10^−2^
3	4.48 × 10^−5^	3.98 × 10^−5^	4.23 × 10^−5^	1.49 × 10^−6^	3.54 × 10^−2^
4	2.58 × 10^−5^	2.34 × 10^−5^	2.46 × 10^−5^	6.49 × 10^−7^	2.63 × 10^−2^
5	4.69 × 10^−5^	4.03 × 10^−5^	4.35 × 10^−5^	2.05 × 10^−6^	4.71 × 10^−2^
6	3.97 × 10^−5^	3.53 × 10^−5^	3.75 × 10^−5^	1.31 × 10^−6^	3.50 × 10^−2^
7	3.96 × 10^−5^	3.46 × 10^−5^	3.72 × 10^−5^	1.46 × 10^−6^	3.94 × 10^−2^
8	5.05 × 10^−6^	4.57 × 10^−6^	4.81 × 10^−6^	1.45 × 10^−7^	3.02 × 10^−2^
9	2.67 × 10^−6^	2.33 × 10^−6^	2.50 × 10^−6^	1.06 × 10^−7^	4.23 × 10^−2^
10	2.12 × 10^−6^	1.88 × 10^−6^	2.00 × 10^−6^	7.00 × 10^−8^	3.50 × 10^−2^
11	5.40 × 10^−6^	4.61 × 10^−6^	5.00 × 10^−6^	2.39 × 10^−7^	4.78 × 10^−2^
12	8.23 × 10^−6^	7.12 × 10^−6^	7.68 × 10^−6^	3.19 × 10^−7^	4.15 × 10^−2^
13	6.29 × 10^−6^	5.28 × 10^−6^	5.78 × 10^−6^	2.92 × 10^−7^	5.04 × 10^−2^
14	9.68 × 10^−6^	8.19 × 10^−6^	8.94 × 10^−6^	4.62 × 10^−7^	5.17 × 10^−2^
15	1.73 × 10^−5^	1.51 × 10^−5^	1.62 × 10^−5^	6.28 × 10^−7^	3.87 × 10^−2^
16	1.26 × 10^−5^	1.16 × 10^−5^	1.21 × 10^−5^	3.19 × 10^−7^	2.64 × 10^−2^
17	2.40 × 10^−6^	2.14 × 10^−6^	2.27 × 10^−6^	8.10 × 10^−8^	3.57 × 10^−2^
18	7.04 × 10^−6^	6.12 × 10^−6^	6.58 × 10^−6^	2.76 × 10^−7^	4.19 × 10^−2^
19	1.08 × 10^−5^	9.86 × 10^−6^	1.03 × 10^−5^	2.59 × 10^−7^	2.52 × 10^−2^
20	2.27 × 10^−5^	1.92 × 10^−5^	2.09 × 10^−5^	1.00 × 10^−6^	4.79 × 10^−2^
21	4.37 × 10^−6^	3.75 × 10^−6^	4.06 × 10^−6^	1.78 × 10^−7^	4.38 × 10^−2^
22	9.75 × 10^−6^	8.44 × 10^−6^	9.09 × 10^−6^	3.80 × 10^−7^	4.18 × 10^−2^
23	3.64 × 10^−5^	3.16 × 10^−5^	3.40 × 10^−5^	1.43 × 10^−6^	4.20 × 10^−2^
24	2.18 × 10^−6^	2.00 × 10^−6^	2.09 × 10^−6^	5.65 × 10^−8^	2.70 × 10^−2^
25	1.07 × 10^−5^	9.89 × 10^−6^	1.03 × 10^−5^	2.25 × 10^−7^	2.19 × 10^−2^
26	1.09 × 10^−5^	9.76 × 10^−6^	1.03 × 10^−5^	3.20 × 10^−7^	3.12 × 10^−2^
27	1.10 × 10^−5^	9.61 × 10^−6^	1.03 × 10^−5^	4.13 × 10^−7^	4.00 × 10^−2^
28	1.12 × 10^−5^	9.47 × 10^−6^	1.03 × 10^−5^	4.76 × 10^−7^	4.60 × 10^−2^
29	1.10 × 10^−5^	9.37 × 10^−6^	1.02 × 10^−5^	4.57 × 10^−7^	4.47 × 10^−2^
30	1.10 × 10^−5^	9.72 × 10^−6^	1.04 × 10^−5^	3.94 × 10^−7^	3.77 × 10^−2^
31	1.09 × 10^−5^	9.64 × 10^−6^	1.03 × 10^−5^	3.95 × 10^−7^	3.84 × 10^−2^

**Table 7:** ANOVA results of tensile strength.

	DF	Adj SS	Adj MS	*F*	*p*
*x*_1_	1	252.89	252.89	7.82	0.013
*x*_2_	1	480.16	480.16	14.84	0.001
*x*_3_	1	1100.49	1100.49	34.02	0
*x*_4_	1	287.7	287.7	8.89	0.009
*x*_1_^2^	1	991	991	30.63	0
*x*_2_^2^	1	668.51	668.51	20.67	0
*x*_3_^2^	1	4.35	4.35	0.13	0.719
*x*_4_^2^	1	130.65	130.65	4.04	0.062
*x*_1_ *x*_2_	1	7.9	7.9	0.24	0.628
*x*_1_ *x*_3_	1	168.02	168.02	5.19	0.037
*x*_1_ *x*_4_	1	12.32	12.32	0.38	0.546
*x*_2_ *x*_3_	1	36.44	36.44	1.13	0.304
*x*_2_ *x*_4_	1	8.69	8.69	0.27	0.611
*x*_3_ *x*_4_	1	3.08	3.08	0.1	0.762
Error term	16	517.59	32.35	-	-

**Table 8:** ANOVA results of electrical conductivity.

	DF	Adj SS	Adj MS	*F*	*p*
*x*_1_	1	7.7 × 10^−5^	7.7 × 10^−5^	1.36	0.026
*x*_2_	1	2.6 × 10^−5^	2.6 × 10^−5^	0.46	0.049
*x*_3_	1	8.9 × 10^−5^	8.9 × 10^−5^	1.58	0.023
*x*_4_	1	2.4 × 10^−3^	2.4 × 10^−3^	42.54	0
*x*_1_^2^	1	1.8 × 10^−6^	1.8 × 10^−6^	0.03	0.086
*x*_2_^2^	1	1.9 × 10^−4^	1.9 × 10^−4^	3.30	0.009
*x*_3_^2^	1	2.4 × 10^−6^	2.4 × 10^−6^	0.04	0.084
*x*_4_^2^	1	2.9 × 10^−4^	2.9 × 10^−4^	5.09	0.004
*x*_1_ *x*_2_	1	1.7 × 10^−4^	1.7 × 10^−4^	2.96	0.010
*x*_1_ *x*_3_	1	4.6 × 10^−5^	4.6 × 10^−5^	0.82	0.038
*x*_1_ *x*_4_	1	1.8 × 10^−4^	1.8 × 10^−4^	3.22	0.009
*x*_2_ *x*_3_	1	1.9 × 10^−4^	1.9 × 10^−4^	3.32	0.009
*x*_2_ *x*_4_	1	9.9 × 10^−5^	9.9 × 10^−5^	1.76	0.020
*x*_3_ *x*_4_	1	1.5 × 10^−5^	1.5 × 10^−5^	0.27	0.061
Error term	16	9.0 × 10^−4^	5.6 × 10^−5^	-	-

**Table 9:** Optimized wet spinning conditions and predicted values.

Optimization		Tensile Strength	Electrical Conductivity	Tensile Strength & Electrical Conductivity
Bath concentration (%)		27.88	20.25	37.03
Bath temperature (°C)		54.48	39.87	58.27
Drying temperature (°C)		130	130	128.1
Elongation (%)		382.83	100	100
Predicted value	Tensile strength (MPa)	68.18	32.78	45.03
	Electrical conductivity (S/cm)	7.31 × 10^−6^	5.84 × 10^−5^	7.31 × 10^−6^

**Table 10:** Experimental values and error values of optimized wet spinning conditions.

Optimization		Tensile Strength	Electrical Conductivity	Tensile Strength & Electrical Conductivity
Experimental value	Tensile strength (MPa)	69.76	33.47	52.53
	Electrical conductivity (S/cm)	5.99 × 10^−6^	5.23 × 10^−5^	1.17 × 10^−5^
Error	Predicted value of Tensile strength	2.3%	2.1%	14.3%
	Experimental value of Electrical conductivity	22%	11.70%	27.4%

## Data Availability

Not applicable.
